# Early procedural training increases anesthesiology residents’ clinical production: a comparative pre-post study of the payoff in clinical training

**DOI:** 10.1186/s12909-021-02693-w

**Published:** 2021-05-06

**Authors:** Claus Hedebo Bisgaard, Svein Aage Rodt, Peter Musaeus, Jens Aage Kølsen Petersen, Sune Leisgaard Mørck Rubak

**Affiliations:** 1 Centre for Health Sciences Education, Faculty of Health, Aarhus University, Palle Juul Jensens Boulevard 82, Building B, DK-8200 Aarhus N, Denmark; 2 Department of Anaesthesiology and Intensive Care, Aarhus University Hospital, Palle Juul-Jensens Boulevard 99, 8200 Aarhus N, Denmark; 3 Centre for Educational Development, Aarhus University, Palle Juul Jensens Boulevard 82, Building B, DK-8200 Aarhus N, Denmark; 4 Department of Anaesthesiology, Aarhus University Hospital, Palle Juul-Jensens Boulevard 99, 8200 Aarhus N, Denmark; 5 Department of Paediatrics and Adolescent Medicine, Center of Paediatric Pulmonology and Allergology, Aarhus University Hospital, Palle Juul Jensens Boulevard 99, 8200 Aarhus N, Denmark

## Abstract

**Background:**

Competency-based education has been shown to enhance clinical skills, improve patient care, and reduce number of complications resulting in a better return on investments. Residents constitute an important workforce at many hospitals. Yet, the effect of training on residents’ contribution to production in patient care is scarcely studied. This study evaluated the effects of early competency-based procedural training on residents’ contribution to patient care in central venous catheterization and spinal and epidural anesthesia.

**Methods:**

The design was a non-randomized cohort study of first-year anesthesiology residents. The intervention group received additional early focused skills training while three control groups received traditional competency-based education. The residents’ contributions to patient care were compared between the intervention group (*n* = 20), a historical control group (*n* = 19), and between a contemporary control group (*n* = 7) and a historical control group (n = 7) from different departments. The residents’ vs specialists’ procedural production share was compared between years within each study group. We calculated specialist time saved compared to the time spent providing additional skills training in the intervention group.

**Results:**

We found statistically significant increases in residents’ vs specialists’ share of total production after the intervention for epidural anesthesia: 2015: 0.51 (0.23, 0.70) to 2017: 0.94 (0.78, 1.05), *p* = 0.011 and central venous catheterization: 2015: 0.30 (0.23, 0.36) to 2016: 0.46 (0.35, 0.55), *p* = .008; and to 2017: 0.64 (0.50, 0.79), *p* = 0.008.

Comparison between residents and specialists on production of the three procedures before and after the intervention showed a surplus of 21 h of freed specialist time per year.

**Conclusions:**

Early procedural training results in more productive residents and freed specialist time for additional supervision, other clinical tasks or research. This provides empirical support for a positive correlation between early focused training and increased independent production among residents.

## Background

In competency-based education (CBE), trainees progress through pre-determined competence levels at an individual pace [1, 2].

Traditionally, medical trainees have been accredited as specialists through completion of a fixed duration of residency supplemented with evaluations by experienced doctors, procedural log books or written examinations [3, 4]. In contrast, CBE curricula define transparent and clinically relevant competence goals for each procedure or skill necessary as a part of a specialist certification [5, 6]. CBE is by some clinicians and researchers perceived as a cornerstone in the continuing professional development (CPD), which is believed to promote quality of health care and focus on patient-relevant outcomes [7]. CBE is thought to optimize learning and depend less on experiences achieved by many patient encounters during an extensive number of working hours [8].

McGaghie and colleagues introduced CBE in medical education in 1978 [9]. Their approach has since been spread to medical education programs internationally and was adopted in Denmark in the late 1990s [10–14]. The competency-based Danish anesthesia training program is divided into a one-year basic residency and a subsequent four-year residency before reaching specialist authorization [15].

Danish anesthesia residents are required to complete 15 so-called competence cards during the first-year residency. These competence cards ensure that the resident obtains sufficient skills and knowledge to be allowed to perform the procedure unsupervised in the expected uncomplicated patient case. The residents are not required to seek supervision for the specific procedure upon completion of the competence cards but are encouraged to do so. Residents complete competence cards in a patient encounter, supervised by a specialist anesthesiologist.

CBE has the potential to speed up competence development due to the individualized learning approach, i.e., learning is paced according to performance. Nevertheless, CBE training programs, including the Danish anesthesia programs, have tended to adhere to the traditional fixed curriculum for clinical post-graduate training [16]. Obtaining competences earlier could result in additional time to gain clinical experience, when the duration of the residency is unchanged. Additional experience would in turn lead to a higher level of skills performance and enhance patient comfort and safety [17–20].

Few studies have investigated the effects of CBE on outcomes such as contribution to clinical production [3, 21, 22]. Long et al. found that neurosurgical residents acquired basic skills earlier after the implementation of CBE compared with the previous traditional clinical training [3]. Van Rossum et al. investigated the financial implications of shortening a clinical training program; they found an overall increase in costs due to the higher salary for specialist-performed procedures [21]. A previous study by our study group investigated if accelerated competence development in basic anesthesia procedures was possible [22].

In contrast, several studies have investigated the effects of CBE in terms of skills level, clinical performance, patient complication rates and return on investment [23, 24]. Not until recently, has trainees’ contribution to production become a research focus, although the costs of training have been in focus for years [21, 25, 26]. The current study combines the clinical production of central venous catheterization (CVC), spinal anesthesia (SA), and epidural anesthesia (EDC) with the additional time spent for early procedural training to evaluate the return on the time invested.

Our hypothesis was that early focused training would result in early achievement of competences in central venous catheterization (CVC), spinal (SA) and epidural anesthesia (EDC), thus leading to increased resident production and freeing of specialist resources.

## Methods

### Study design

The study was a non-randomized prospective intervention study with contemporary and historic control groups.

### Recruitment

All participants were recruited by e-mail. Participants were informed of voluntary participation, anonymity, and the possibility to withdraw from the study at any time.

The inclusion criteria were initiation of Danish first-year anesthesia residency at one of the participating departments from January 1, 2013 to October 1, 2016.

Exclusion criteria were a previous anesthesiology residency or other anesthesiology employment to minimize the risk of difference in existing procedural experience prior to the residency.

### Allocation

#### Intervention group

Four out of five departments of anesthesiology involved in basic residency training for post-graduate education in North Denmark Region participated in the intervention group; these departments are referred to as the intervention departments. First-year residents in the intervention group commenced their one-year residency from January 1, 2015 until October 1, 2016. The intervention group residents were informed by e-mail of the accelerated deadlines for basic procedural competence development and the additional skills training.

The departments all accepted the intervention protocol. Additionally, they already used mannequin-based skills training in their residency training, thus minimizing the influence of unfamiliarity with the mannequins. Finally, we sought to minimize the risk of contamination from one training regime to another by keeping residents from each study group at separate departments.

#### Control group 1

Residents initiating residency training from January 1, 2013 to December 31, 2014 at the intervention departments, served as the historic controls for changes in production from before to after the intervention, thus indicating an effect from the intervention.

#### Control group 2

The anesthesiology departments in this group were located at one hospital in the post-graduate medical education program in North Denmark Region and three hospitals in the Region of Southern Denmark; henceforth referred to as the control departments. Residents in this control group started their residency between January 1, 2015 and October 1, 2016, the same time as the intervention group. Control group 2 trainees were informed of the study’s focus on competence development and clinical production during the first year of residency. In this way, they served as controls of the influence of knowledge of study focus on the result, a type of Hawthorne effect.

#### Control group 3

This group included first-year anesthesia residents from the control departments group starting their residency from January 1, 2013 to December 31, 2014, the same time as the control group 1. They served as the same control for changes in production before and after the intervention at the control departments.

The participants in the control groups were not informed of deadlines or interventions during their training.

All control group residents received traditional CBE training and were expected to complete the competence cards at the intervals defined by the national first-year residency curriculum [15].

Departments for all study groups provided clinical supervision in addition to mannequin-based procedural skills training. This training was the same before and during the intervention in each study group.

Inclusion criteria were completion of residency and availability of data for the first year of residency. Residents on maternity leave were excluded.

## Data sources

### Database data

We retrieved data on the number of performed CVC, SA and EDC from the electronic patient records in Central Denmark Region and the Danish Anesthesia Database. Procedural production data for all first-year residents were available and used in the calculations.

Registrations in the electronic patient records are legal documents in line with other patient records, and the identity of the health professional performing a procedure is registered. The Danish Anesthesia Database is a quality database, primarily used for quality control and research purposes, and functions as an add-on to data recorded in the patient records. Those performing procedures are registered anonymously to eliminate the risk of identification and thereby increase the likelihood of full reporting of errors. Thus, we could not identify the individual performing the procedure, only the educational level such as residency or specialist.

We chose to compare data before and after the intervention at intervention and control departments, respectively. By comparing data from the same databases over time, we attempted to eliminate the risk of changes occurring due to differences in registration practices.

For the calculation of time-related return on investment, we only used data from the intervention departments as residency training at the control departments was unchanged during the intervention period.

The central registers at the post-graduate medical education programs in North and Central Denmark Region and Region of Southern Denmark provided the start and end dates of first year residents during the study period as well as information on those granted maternity leave.

The public register for health professionals’ registration at the Danish Patient Safety Authority was accessed for information on the dates when specialist accreditation was obtained.

### Intervention

Twenty-one competences must be obtained to complete the one-year basic residency. These competences comprise both practical skills and theoretical knowledge relevant to the performance of skills and procedures.

The residents and the supervisors at the intervention departments were instructed to complete six of these competence cards 6 to 24 weeks earlier than previously required in accordance with the standard education protocol. The participating departments agreed to provide a minimum of 4 h of skills training to enable residents to complete the competences within protocol deadlines. A one-day, full-scale simulation course in general anesthesia scenarios was offered to the intervention group residents in the third or fourth week of residency. The interventions and proposed protocol deadlines of competence cards are shown in Table 1.

**Table 1:** interventional overview

Competence card (no.) Part task Trainer	Skills training minimum	Additional training	Protocoled deadline
**Airway management (1)**	2*30 min	One-day simulation course Clinical training	6 weeks
**Anesthesia device (2)**		One-day simulation course Clinical training	6 weeks
**General anesthesia, GA, uncomplicated elective patient (3)**		One-day simulation course Clinical training	6 weeks
**Spinal anesthesia (5)**	2*30 min	Clinical training	9 weeks
**Epidural Block (6)**	2*30 min	Clinical training	9 weeks
**Central venous Catheter, CVC (7)**	2*30 min	Clinical training	12 weeks

Procedural skills training at the departments was performed using procedural simulators. A final year resident or an anesthesiology specialist supervised the simulation-based skills training sessions in all study groups. The procedural simulators used for the skills training were:

For airway management: Airway Management Trainer (Laerdal® Medical, Stavanger Norway).

For spinal and epidural anesthesia: Lumbar Puncture Simulator II M43B (Kyoto Kagaku Co., Ltd., Japan).

For central venous catheterization: Ultrasound CVC Insertion Simulator II (Kyoto Kagaku Co., Ltd., Japan).

The first author instructed residents at the supplemental simulation day on general anesthesia in the third or fourth week of residency. This simulation day consisted of short-dialogue workshops concerning general anesthesia followed by simulations of general anesthesia using SimMan® (Laerdal Medical AS, Stavanger, Norway) in a fully equipped simulated operating room.

The five full-scale simulation scenarios increased in difficulty and covered uncomplicated induction of general anesthesia, rapid sequence induction, unexpected difficult airway management, insufficient anesthesia in prone position, and insufficient ventilation due to intra-tracheal cuff leak. Following a short briefing regarding patient history and planned surgical technique, the trainees briefly discussed the anesthetic strategy before engaging in the scenario. The scenarios and the subsequent debriefings were facilitated by the first author using the Steinwachs model: Description, Analysis/Analogy, and Application [27].

Both intervention and control departments provided additional clinical supervision of individual competences. This clinical supervision was the same over time for all study groups. Control groups residents also received simulation-based education in addition to the clinical supervision, though not in the same focused method as the intervention group.

First year residency duration was unchanged, 1 year, for all study groups, thus allowing for comparison of same duration for contribution to clinical production.

The competence cards were eligible for approval when the resident felt ready for accreditation. Accreditation was obtained when possible, in a clinical setting by specialist assessment in accordance with the specific competence cards.

### Statistical analyses

The Statistical Package for Social Sciences, SPSS by IBM Corporation, USA was used for the statistical analysis.

We calculated the monthly shares of total clinical production for both residents and specialists for central venous catheterization, spinal and epidural anesthesia. We pooled these results into years and subsequently compared the shares using the equation:

**R/S = (resident production/total production) / (specialist production/total production).**

We used Friedman’s test for related medians to calculate the difference between years and quantified significant pairwise comparisons using the Wilcoxon rank test [28, 29].

The effect size (r) was calculated by the equation: r = Z/√N, in which Z is the z-value of the Wilcoxon rank test and N the total number of samples.

For the intervention departments, we then calculated if specialist work time was saved due to increased resident contributions to the total production. We chose the time span between November 2015 and October 2016 as the intervention year, because it represented 12 months of solely intervention group resident employment at the intervention departments. We used 2014 as reference year because it was the last year with no intervention group residents, thus creating the shortest temporal division between the years compared.

The specialist time spent performing simulation skills training in the departments was estimated as the minimum time defined in Table 1. This represents the time spent in actual training and the subsequent evaluation. Preparation time is not included in this estimate, as the instructors were experienced in the specific simulation skills training modules and thus in need of very little preparation, both before and after the intervention period.

We used a seven-step statistical process to account for differences in total production and the number of residents employed between years:

1. We calculated a correctional factor for differences in resident employment, “Resident factor”, by dividing the mean number of residents employed per month in 2014 by the values from the intervention year.
2. We corrected for differences in total production, “Procedural factor”, by dividing the total number of CVCs, SAs and EDCs performed in 2014 by the procedures performed in the intervention year.
3. We subtracted residents’ production from total production in the reference year to obtain the specialist production of the reference year.
4. We calculated standardized specialist production by first multiplying the actual residents’ production from the intervention year by the resident factor and then subtracting this number from the intervention year total production. This result corresponds to the theoretical specialist production given the number of residents was the same in the intervention and reference year, respectively.
5. We calculated the difference in specialist annual production by subtracting the standardized specialist production from the specialist production of the reference year, then multiplying the difference by the procedural factor. The result is the theoretical specialist production if the total production of the intervention year was the same as the intervention year.
6. For each procedure, we calculated the difference in specialist time spent performing the procedures annually for each procedure by multiplying the difference in specialist annual production by estimated specialist time spent performing the procedure.
7. We subtracted the aggregated time difference for all three procedures from the time invested in skills training to create an estimated return on time invested in supervision.

The calculations and results are shown in Table 2.

**Table 2:** Specialists’ procedural time saved

	Reference year 2014	Intervention year Nov 2015-0ct 2016	Resident Correction Factor			
**Employed residents, mean, (95% CI)**	17.4, (16.6, 18.9)	15.1 (13.1, 17.1)	1.15			
	**Specialist production** **No of procedures**	**Specialist production** **No of procedures**	**Procedural** **Correction Factor**	**Standardized** **Specialist production** **No of procedures**	**Difference in specialist production** No of procedures	**Time** Minutes
**Spinal Anesthesia**	4319–720 = 3599	4052–587 = 3465	1.07	4052-(587*1.15) = 3376	(3376–3599) * 1.07 = 239	−239 * 10 = − 2390 min
**Epidural Anesthesia**	3144–881 = 2263	3302–693 = 2609	0.95	3302-(693*1.15) = 2503	(2503–2263) * 0.95 = 229	229 * 15 = 3435 min
**Central Venous Catheter**	1879–344 = 1535	1564–289 = 1275	1.20	1564-(289*1.15) = 1231	(1231–1535) * 1.2 = − 365	−365 * 15 = − 5475 min
**Total**						3435–7865 = - 4430 min, or 73 h saved
**Return on time invested**				17.4 * 3 h = 52.2 h		52–73 h = 21 h saved

Resident Correction Factor” = Mean monthly employed residents for Reference year/Intervention year

“Procedural Correction Factor” = Total specialist procedural production for reference year/intervention year

Specialist production = Total procedural production for departments-residents production

Standardized Specialist Production = Intervention year total production-(intervention year residents’ production*resident correction factor)

Difference in specialist production = (Standardized Specialist production-Reference year specialist production)*Procedural Correction Factor

Return on time invested = Difference in Specialist Production*Estimated Specialist time spent for performance of each procedure

### Ethics

Ethics approval was not required according to the Central Denmark Committee on Health Research Ethics (case no: 1–10–72-113-14) as no patient referable data were involved. Approval for the use and protection of production database data was obtained from the Danish Data Protection Agency (journal numbers 2015-57-0002 and 62,908).

## Results

**Fig. 1:**
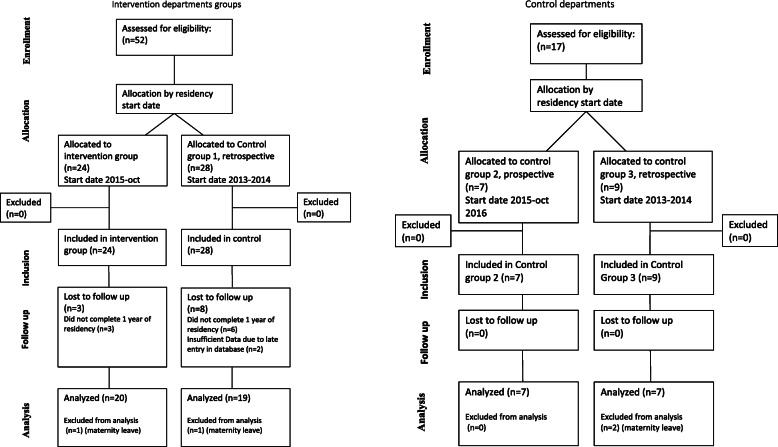
Inclusion results

The inclusion process for the residents is outlined in Fig. 1.

The number of residents in control groups 2 and 3 was much lower because only data from one department were available for analysis.

Demographic data from the four groups are presented in Table 3.

**Table 3:** Participant demographics

		Intervention	Control group 1	Control group 2	Control group 3
**Participants (n)**		20	19	7	7
**Age**	*Mean, CI [,]*	30.5 [29.6, 31.3]	31.3 [30.5, 32.1]	29.9, [28.0, 32.1]	31.4, [29.6, 33.6]
**Gender**	*Male*	7	10	4	2
	*Female*	13	9	3	5
**PGY**	*Mean, CI [,]*	2.0 [1.6, 2.3]	2.3 [1.8, 2.8]	1.9, [1.1, 3.5]	3.4, [1.8, 3.5]
**Pre. intro**	*Yes*	10	7	1	1
	*No*	10	12	6	6

Bias-corrected and accelerated bootstrap (1000 samples)

*PGY* PostGraduate Year; *CI* Confidence Interval

No significant differences were found in the demographic data at baseline.

Boxplots 1–6 show the results when comparing the residents’ share of the total annual production divided by the share of total production performed by specialists. The boxplots show the median for each year, each boxplot illustrating one procedure for one region. In this way, the development over time as well as the time for pre- and post-intervention is visualized. All procedures showed an increase after the intervention was initiated in 2015 to the end of the intervention in 2017 for the intervention departments. This development was not seen for the control departments.

Testing for significant differences between the years within the same regional groups are listed in Table 4. Significant differences were found in pairwise comparisons between the pre- and post-intervention years for EDC 2015 to 2017, effect size *r* = 0.60, *p* = 0.01, and CVC 2015 to 2017, *r* = 0.62, *p* < 0.01, and CVC 2016 to 2017, *r* = 0.62, *p* < 0.01. Wilcoxon effect sizes are defined as small (*r* < 0.3), moderate (0.3 < *r* < 0.5), and large (*r* > 0.5) [29].

**Table 4:** Differences and effect sizes in residents’ vs specialists’ share of total production

Comparison	Test statistic, T	Effect Size, r	***p***-value
**Spinal 2013 vs 2015**	4	−0.56	.006
**Spinal 2013 vs 2016**	2	−0.59	.004
**EDC 2013 vs 2015**	6	−0.53	.010
**EDC 2013 vs 2016**	0	−0.54	.002
**EDC 2015 vs 2017**	44	0.60	.011
**CVC 2013 vs 2015**	12	−0.43	.034
**CVC 2015 vs 2017**	0	0.62	.008
**CVC 2016 vs 2017**	0	0.62	.008

Finally, the specialists’ annual procedural time and additional supervision time before and after the intervention were compared (Table 2). Overall saving of specialist working time was 21 h, indicating a positive net result of introducing early focused residency training.

## Discussion

### Primary findings

The current study showed that early procedural training increased residents’ contribution and reduced specialists’ contribution to total annual production of SA, EDC and CVC. The time invested in additional supervision was cost-effective and translated into a surplus of 21 h of freed specialist time. This surplus of specialist time allowed time for additional supervision enabling residents to obtain a higher level of proficiency or acquire additional skills. The specialists performing the supervised training sessions are also clinicians thus specialist time saved may be used for more advanced clinical tasks. This effect was likely caused by the intervention because corrections for fluctuations in the number of employed residents and annual production were taken into consideration in our analyses.

The share of CVCs inserted by intervention residents increased in both 2016 and 2017, and EDCs were increased in 2017 compared to 2015. An increasing share of the intervention trainees were intervention group residents in 2015 and almost all of the trainees were intervention trainees in 2016; this share declined in 2017 after inclusion ended in October 2016. However, the positive effect on CVC production seemed to be maintained, indicating an effect beyond the intervention. This positive effect from 2015 to 2016 and 2017 was also seen for SA, though this effect was not statistically significant.

The positive results in our study are in line with several earlier studies showing positive translational effects from CBE. Enhanced clinical performance, reduced patient complications rates, and derived positive cost-benefits have all been linked to short duration CBE programs [23, 30].

Only one previous study has investigated the cost-benefit of a CBE program from the perspective of the trainees’ contributions to clinical production; this study showed higher total costs as a result of a shorter residency despite a reduced salary expenditure [21]. The deficit in revenue derived from the reduced resident production due to the shorter duration of residency. In contrast, the intervention group in our study maintained the same fixed residency duration despite the acceleration in competency training. This enabled the same time for contribution to production, addressing some of the challenges in terms of the unpredictability of progression and clinical workforce capacity [16].

### Strengths and limitations

A strength of the study is the database used for the primary comparison of data among intervention and historical residents. The data from the electronic patient records in Central Denmark Region are legal documents and subject to great care of registration. Thus, the procedure counts from this database have a high face validity.

There may be a risk of registration bias in the registration of the performer of a specific procedure. However, there were no differences in registration practices between residents and specialists in the study period. Additionally, the potential bias of using two databases with different purposes has little effect on the primary results because we compared different years at the same departments using the same databases.

We did not hypothesize the relatively high share of residents’ production in 2013 compared with all subsequent years. A shift in residents’ learning focus from spinal anesthesia towards peripheral nerve blocks was seen as a possible confounder. Subsequent calculations on total number of blocks performed by first-year residents showed an increase from a low number in 2013 to a high in 2017. This shift in focus could also account for the increase in specialist-performed EDCs in the intervention year of the return on time calculation. As the study focused on three selected procedures, there is a risk of overlooking a shift in residents’ focus towards other procedures.

There were no available records of the actual hours of skills training performed. We therefore estimated that the time invested in supervised skills training was as described in the study protocol (Table 1). Similarly, we did not have access to registers of the actual work hours completed by the residents; thus, weekly work hours have been estimated as 37 h per week in accordance with Danish legislation. Both estimates represent possible information bias influencing the return-on-investment calculation. Additional unrecorded specialist time spent on supervising skills training would reduce the calculated return on time due to increased investment and a more rigid registration of these supervision hours would have been desirable. Increases in work hours between study groups would result in increases in opportunities to perform the procedures, thus leading to a larger return on invested time. Due to strict budgetary requirements, the 37-h workweek has been upheld throughout the study period and it is thus of minor importance.

The results of accelerated competence completion described in a previous paper by our group shows variance in time before completion of competence cards between learners [22]. There is a risk of fast learners profiting mostly from this acceleration of competence formation while slower learners will benefit less or be negatively affected by the intensive learning environment. The increase in procedural production of the present study suggests that there is a positive effect even if variation in learning aptitudes exists.

As the primary focus was on the quantity of procedures, there might be an increased risk of not acknowledging a difference in the quality of the procedures performed upon completion of the competence cards. The competence card completion are supervised procedures followed by a standardized supervised evaluation of a clinical performance and theoretical exam. This standardization is thought to ensure uniformity of the minimum skill level and quality ahead of independent performance. For the subsequent independent procedural performance, we have no information of the quality of procedural performance or the need for supplemental supervision. As such, we cannot estimate a possible change in quality caused by early, focused training.

### Future research and educational perspectives

This study is the first to link an educational intervention to clinical production. The calculated surplus of specialist working hours derived from the increase in residents’ production indicates a positive trade-off from early focused resident training. Even as the calculated surplus hours are relatively few and might be subject to confounders, we see the result as a strong argument for early educational focus as a way of diminishing daily department workload.

Although the current study only focuses on three specific procedures, other procedures and competences may have similar potentials. Extrapolating to the remaining first year Danish anesthesia training program, a total of 15 competences are required for completion, indicating further potential for saved time. Expanding the scope, the four-year senior anesthesia residency encompasses a further 20 competence cards, adding even further time savings from such an intervention. On an even a larger scale, CBE has been introduced in several other specialties in Denmark and worldwide. There might be a great educational potential for re-organizing the use of residents’ and specialists’ time working and learning together. Furthermore, the specialists could spend more time on supervision, and this would presumably improve the residents’ production and education. Further studies in different settings are needed to evaluate these effects.

## Conclusions

This study demonstrates that accelerated training in first-year anesthesia residency leads to a higher productivity among residents in the areas of spinal anesthesia, epidural anesthesia and central venous catheterization. The positive results on specialist time invested in supervising the residents provide an important argument for not considering graduate medical education as a burden. Accelerated training can produce relevant clinical outcomes and is thus a sound investment.

## Data Availability

The datasets used and/or analyzed during the current study are available from the corresponding author on reasonable request.
